# Chest X-ray findings in HIV- infected Highly Active Antiretroviral Treatment (HAART)-naïve patients

**Published:** 2012-07-19

**Authors:** Akinsegun Akinbami, Babajide Balogun, Modupe Balogun, Owolabi Dosunmu, Olajumoke Oshinaike, Adewumi Adediran, Kayode Adegboyega

**Affiliations:** 1 Department of Haematology and Blood Transfusion, Lagos State University College of Medicine, Nigeria; 2 Department of Radiology, Lagos State University College of Medicine, Nigeria; 3 Department of Internal Medicine, Lagos State University College of Medicine, Nigeria; 4 Department of Haematology and Blood Transfusion, College of Medicine, University of Lagos, Nigeria

**Keywords:** Chest X-ray, HIV-infected, HAART-naïve

## Abstract

**Introduction:**

Patients with human immunodeficiency virus (HIV) infection frequently present with a wide spectrum of pulmonary and cardiac complications from the virus, opportunistic infections and neoplasms that may be associated with a high mortality rate. Diseases of the respiratory tract account for about half of deaths from AIDS, while cardiac diseases account for more than a quarter of deaths from AIDS. This study aimed at determining the prevalence of pulmonary and cardiac diseases using a chest radiograph in HAART-naïve HIV-infected patients.

**Methods:**

This study was conducted at Lagos State University Teaching Hospital (LASUTH) HIV clinic between September 2010 and August 2011 amongst all registered HAART-naïve HIV/AIDS patients. Patients had posterior-anterior chest radiographs done in full inspiration. Participants were asked and aided to fill the structured questionnaires to obtain demographic data.

**Results:**

Out of a total of one hundred and two recruited for the study, 54 ( 52.94%) had a normal chest radiograph, while 48 (47.06%) had abnormal chest radiograph .The abnormal findings included, 27.45% who had bronchopneumonia, 6.86% cardiomegaly, 5.88% pulmonary tuberculosis, 5.88% radiological features of congestive cardiac failure, and 0.98% bronchitis.

**Conclusion:**

It appears that more than half of HAART–naïve HIV-infected patients have normal chest radiographs. Bronchopneumonia (27.5%) is the commonest pulmonary abnormality associated with HIV infection, while the prevalence of pulmonary tuberculosis is 5.88%.

## Introduction

HIV- infected highly active antiretroviral treatment (HAART) -naïve patients are groups of HIV-infected who have not been exposed to antiretroviral drugs either because they have not met the criteria for initiation of therapy laid down by the World Health Organization (WHO) [1], or newly diagnosed patients undergoing pre-treatment evaluation. Diseases of the respiratory tract account for about half of deaths from AIDS [2]. An important diagnostic tool in assessing the respiratory complications as well as the manifestations of HIV infection is the chest x-ray[3]. Pulmonary disorders remain an important complication of HIV infection, even in the current era of potent antiretroviral therapy. Recent findings indicate that bacterial pneumonia and acute bronchitis are currently the most common causes of respiratory disease in HIV-infected individuals in developed countries and are frequently the first clinical manifestation of HIV-infection [3]. The spectrum of diseases of the chest associated with HIV infection is vast and varied. These include a wide spectrum of pulmonary complications from opportunistic infections and neoplasms that may be associated with a high mortality rate. In the developed world, postmortem and echocardiography studies suggest that the prevalence of HIV-associated cardiomyopathy in the pre-HAART era was 30% to 40%, and the annual incidence was 15.9 per 1000 patients [4]. In a study to determine the aetiological agents and presentation of HIV/AIDS-associated pulmonary infections in patients presenting for bronchoscopy, Kibiki et al[5] reported that common bacteria were identified in 29.2% of the patients, mycobacterium tuberculosis in 23.3%, human herpes virus 8 (HHV8) in 10%, pneumocystis jiroveci in 7.5% and fungi in 4.2% patients. Mycobacterial pulmonary infections in AIDS are most commonly caused by *Mycobacterium tuberculosis*. Non tuberculous mycobacteria i.e. *Mycobacterium avium* complex (MAC), *Mycobacterium kansasii* and *Mycobacterium fortuitum*, are seen infrequently. Radiographically, the findings of tuberculosis in AIDS patients are similar to those of nonimmunocompromised patients, with nodular densities and cavitation of larger nodules, mainly in upper lobes.

*Pneumocystis jiroveci* pneumonia is one of the most frequent and severe opportunistic infections in patients with AIDS. Over half of all AIDS patients will have at least one episode of *Pneumocystis jiroveci* pneumonia at some point during their clinical course, with mortality from a single episode ranging from 10 to 30% [6]. Radiographically, the findings with *Pneumocystis jiroveci* pneumonia are quite variable. Chest x-ray may be normal early in the course of the disease. As *Pneumocystis jiroveci* pneumonia progresses there may be interstitial or reticulonodular infiltrates, nodules, or even patchy areas of consolidation. *Pneumocystis jiroveci* typically produces a pneumonia that is widespread throughout the lungs [7].

In AIDS, toxoplasmosis is usually associated with disseminated infection and secondary pulmonary involvement. The most common clinical finding is cough, which may be either productive or non-productive. An abnormal chest roentgenogram marked by bilateral interstitial infiltrates may appear in only half of cases. Diagnosis can be made by bronchoalveolar lavage in most cases [8]. Bacterial pneumonias in AIDS can lead to significant morbidity and mortality and are second only to *Pneumocystis jirovecii* pneumonia as an immediate cause of death [2]. Overall, bacterial organisms account for more pulmonary infections than other infectious agents in persons with AIDS [9]. Acute bronchopneumonia may be suggested by bronchoalveolar lavage or transbronchial biopsies in which neutrophilic exudate is present and gram stain reveals bacteria. Bacterial bronchopneumonias may also be present along with other opportunistic infections [10].

Aside from cytomegalovirus, other viral infections of the lung are less frequently diagnosed, though the true incidence remains unknown. Viruses may be recovered from bronchoalveolar lavage fluid. Viral pneumonitis may be difficult to distinguish from nonspecific interstitial pneumonitis or lymphoid interstitial pneumonitis without specific viral cultures or serologies. Bacterial infections often complicate viral pneumonitis and may be indistinguishable clinically, though a viral pathogen may be the only infectious agent present in some cases. Viral pneumonias most frequently are due to herpes simplex, rhinovirus, influenza, parainfluenza, and adenovirus in adults, with respiratory syncytial virus more frequent in children. Mycoplasma species, though not viruses, can produce a similar clinical picture with infection, and can also be recovered with bronchoalveolar lavage [10].

The diseases of the respiratory tract account for about half of deaths from AIDS, [2] while cardiac diseases account for more than a quarter of deaths from AIDS [4]. The need to determine the pattern of cardiac and pulmonary diseases in HIV/AIDS patients with a view to giving specific intervention cannot therefore be overemphasized. Chest radiographic pathologies are common, and the nature and distribution of pulmonary and cardiac findings on the chest radiograph will often suffice in suggesting a diagnosis or differential diagnosis. Most chest diseases will be associated with abnormal chest radiographic findings. If the radiograph is normal or equivocal and clinical suspicion for disease is high, computer tomography scan (CT scan) should be performed to evaluate for subtle pulmonary and cardiac abnormalities. The objective of this study is to provide the radiological features seen on chest radiographs of treatment- naïve HIV patients seen at Lagos State University Teaching Hospital (LASUTH) HIV clinic between September 2010 and August 2011.

## Methods

This study was conducted at Lagos State University Teaching Hospital (LASUTH) HIV clinic between September 2010 and August 2011 amongst all registered HAART-naive HIV/AIDS patients. Chest radiograph is a pre-treatment evaluation done by all registered HIV patients at LASUTH free of charge. A total of one hundred and two consenting participants were recruited consecutively into the study. All had postero-anterior (PA) chest radiographs done in full inspiration in the radiology department. Chest radiographs were read and quality evaluated by the radiologists in the team. Participants were asked and aided to fill the structured questionnaires to obtain demographic data, radiological characteristics evaluated in the radiographs are shown in the questionnaire. (Appendix 1). The inclusion criteria were adult patients who tested positive to HIV antibody and yet to commence highly active antiretroviral treatment. An exclusion criterion was HIV-infected patients already on HAART. Approval for research work was obtained from the institution's research and ethical committee.

### Statistical Analysis

Data were analyzed using SPSS version 16.0 (Statistical Package for Social Sciences, Inc., Chicago, Ill); a statistical computer software. The descriptive data were given as means ± standard deviation (SD). The differences were considered to be statistically significant when the p value obtained is less than 0.05.

## Results

A total of 102 patients were recruited for the study consisting of 61 (59.80%) females and 41 (40.19%) males (Table 1). The mean age was 37.72±9.11 years, with a minimum of 23 years and maximum of 64. More than half, 55 (53.92%) were symptomatic, while 47 (46.07%) were asymptomatic. Forty-five (44.11%) of all patients had cough as symptom, out of which 40 of them (88.88%) had productive cough.

**Table 1:** Age distribution of patients

	Gender		Total
Age (Years)	Male	Female	
23-27	0	12	12
28-33	4	21	25
34-39	12	10	22
40-45	18	9	27
>45	7	9	16
**Total**	**41**	**61**	**102**

Twenty-six (25.49%) of all patients had chest pain, while twelve (11.76%) had dyspnea. Sixty-four (62.74%) of patients experienced weight loss, while and 32 (31.37%) had night sweat.

More than half, (Table 2) 54 (52.94%) had a normal chest radiograph, while 48 (47.06%) had abnormal chest radiograph .The abnormal findings included, 28 of 102 (27.45%) having bronchopneumonia, 7 (6.86%) cardiomegaly, 6 (5.88%) pulmonary tuberculosis, 6 (5.88%) radiological features of congestive cardiac features, and 1 (0.98%) bronchitis.

**Table 2:** Radiological diagnoses based on radiographic findings

	Frequency	Percent
Normal chest	54	52.94
Cardiomegaly	7	6.86
Bronchopneumonia	28	27.45
Bronchitis	1	0.98
Pulmonary Tuberculosis	6	5.88
Congestive Cardiac failure	6	5.88
**Total**	**102**	**100**

The diagnoses were arrived at by finding the following radiological patterns in the radiographs, cavity lesions and fibrotic streaks which were located in the right lung field in 6 of 102(5.88%) of the patients (tuberculosis), while 28 (27.45%) of the patients had patchy opacities scattered in all the zones (bronchopneumonia). Seven (6.86%) had increased cardiothoracic ratio (cardiomegaly). Only 1 (0.98%) of the patients had bronchitis (bronchial wall thickening).Upper lobe blood diversion (congestive cardiac failure) was seen in 6 (5.88%) patients.

## Discussion

A total of 52.94% of the study population had a normal chest radiograph in this study compared with 32% reported by Adeyekun and Onunu [11] in 2002. The chest radiograph may be normal in HIV- infected patients despite active infection because of their weak immunity that could not mount a granuolomatous reaction to invading organism [12]. The disparity in the prevalence of normal chest radiographs in the two studies may be associated with the different degrees of immunosupression of the two study populations (which were not considered in the two studies). The lower the CD4 count of the study population the more predisposed to opportunistic infections of the lung and the more the tendency for the chest radiograph findings to be atypical[13]. The hallmarks of emergence of HIV infection from clinical latency are a marked decline in the CD4 lymphocyte count and an increase in viraemia. Replication of HIV increases as the infection progresses. There is loss of normal lymph node architecture as the immune system fails. The development of signs and symptoms of AIDS typically parallels laboratory testing for CD4 lymphocytes. A decrease in the total CD4 lymphocyte count below 500/microliter presages the development of clinical AIDS, and a drop below 200/microliter not only defines AIDS, but also indicates a high probability for the development of AIDS-related opportunistic infections and/or neoplasms. The risk for death from HIV infection above the 200/microliter CD4 level is low [14–16].

Majority of patients (27.4%) with abnormal chest radiograph in this study had bronchopneumonia which is in keeping with the study of Kibiki et al[5] in Tanzania. It is also consistent with the study reported by Aviram G.et al. in developed countries [3]. The neutropenia that may occur with AIDS, either as a consequence of HIV infection or as a complication of drug therapy, significantly increases the risk for bacterial infections when the absoulte neutrophil count diminishes below 750/microliter, and particularly when the count is below 500/microliter [16]. Bacterial bronchopneumonia is second only to *Pneumocystis jiroveci* pneumonia in frequency as a cause of death from pulmonary infections in persons with AIDS among caucasians [2].

Bacterial organisms in persons with AIDS most often produce respiratory disease, particularly bronchopneumonia that can be life-threatening, but such infections can become disseminated as well, and recurrence is common. The bronchopneumonias seen with AIDS can be extensive and bilateral. Mortality is higher than in non-HIV-infected patients. Bacterial septicemias are the immediate cause of death in about 5% of AIDS patients [17].

A prevalence of 5.8% was noted for pulmonary tuberculosis in this study. This is contrary to 15.4% reported in Benin City Nigeria [11], 23.3% in Kilimanjaro, Tanzania[4] and 26% in New York, USA [18]. This may also be associated with the different degrees of immunosupression of the study population. However, it is known that about 15% of AIDS patients with tuberculosis will have a negative chest roentgenogram, while this rarely occurs in non-AIDS patients [17].

Cardiomegaly of 6.86% was observed in the study and 5.88% had features consistent with congestive cardiac failure. Cardiac manifestation in HIV infection results from myocardial invasion with HIV itself, autoimmune response to viral infection, nutritional deficiencies, opportunistic infections, and prolonged immunosupression. Clinico-pathological studies from the pre-HAART era showed a 30% prevalence of cardiomyopathy in patients with AIDS. It is associated with a poor prognosis, and results in symptomatic heart failure in up to 5% of HIV patients [19].

## Conclusion

It can be concluded from this study that while more than half of HAART–naïve HIV patients have normal chest radiographs, bronchopneumonia is the commonest abnormality associated with HIV infection, and the prevalence of pulmonary tuberculosis and cardiac diseases is relatively low. Based on the findings from the study, the use of chest radiograph should be mandatory for all HIV-infected patients, but its use alone may not be totally adequate in elucidating the vast and varied microorganisms associated with pulmonary and cardiac diseases in HIV/AIDS patients because of its limitation.

**Limitation of the study:** Reliability on chest-x-ray findings in making diagnoses of pulmonary infections without the use of laboratory investigations for confirmation.

## Additional material

**Table 1:** Questionnaire
